# B chromosomes in the species *Prochilodus argenteus* (Characiformes, Prochilodontidae): morphologicalidentity and dispersion

**DOI:** 10.3897/CompCytogen.v9i1.8587

**Published:** 2015-03-20

**Authors:** Manolo Penitente, Fausto Foresti, Fábio Porto-Foresti

**Affiliations:** 1 Departamento de Ciências Biológicas, Faculdade de Ciências, Universidade Estadual Paulista (Unesp), Campus de Bauru, Bauru, SP, Brazil; 2 Departamento de Morfologia, Instituto de Biociências, Universidade Estadual Paulista (Unesp), Distrito de Rubião Junior, s/n, 18618-970, Botucatu, SP, Brazil

**Keywords:** Curimbatá, cytogenetic, neotropical fish, supernumerary chromosomes

## Abstract

B chromosomes have attracted the attention of Neotropical fish cytogeneticists in recent years, both for their remarkable occurrence in this group and also because of the interest in studies of the genetic structure and role played in the genome of these organisms. The aim of this study was to report the first occurrence of supernumerary chromosomes in *Prochilodus argenteus* (Agassiz, 1829), this being the fifth carrier species among thirteen within the genus *Prochilodus* (Agassiz, 1829). The extra elements identified in this species are small sized heterochromatic chromosomes characterized by a low mitotic instability index, being very similar to other supernumerary chromosomes described in the species of the genus *Prochilodus*. Morphology, structure and dispersion of the supernumerary genomic elements which occur in species of this genus are discussed aiming to better understand aspects involved the origin of supernumerary chromosomes and the differentiation process and relationships among species of this family.

## Introduction

Among the Characiformes fish, representatives of the family Prochilodontidae are distinguished by their abundance in the environments in which they occur, by their wide distribution in South America and the high migratory capacity displayed by the species of this group of organisms (Castro 1990, Sivasundar et al. 2001, Turner et al. 2004). According to Castro and Vari (2004) the family Prochilodontidae consists of three genera: *Prochilodus* (Agassiz, 1829), *Semaprochilodus* (Fowler, 1941), and *Ichthyoelephas* (Posada Arango, 1909), which include thirteen, six and two species, respectively. This family can be easily distinguished from other Characiformes families through a distinct set of morphological characters (Castro 1990), and fishes of the genus *Prochilodus* can be highlighted as a pioneer group in studies of B chromosomes among Neotropical fishes.

Besides the conservative karyotype formulae found among representatives of the genus *Prochilodus*, variation in the chromosome number can be observed among species due to the occurrence of supernumerary chromosomes. Among seven species of this genus, the occurrence of B chromosomes has been identified and described in four species, present in *Prochilodus lineatus* Valenciennes, 1836 (Pauls and Bertollo 1983), *Prochilodus nigricans* (Agassiz, 1829) (Pauls and Bertollo 1990, Venere et al. 1999), *Prochilodus brevis* (Steindachner, 1874) (Pauls and Bertollo 1990) and *Prochilodus mariae* Eigenmann, 1922 (Oliveira et al. 2003), but not occurring in cells of *Prochilodus argenteus* (Agassiz, 1829), *Prochilodus costatus* (Valenciennes, 1850) and *Prochilodus vimboides* (Kner, 1859), as described by Pauls and Bertollo (1990).

In this paper we describe for the first time the occurrence of supernumerary chromosomes in *Prochilodus argenteus*, identifying its similarity with the extra genomic elements characteristic of this group. The description of the occurrence of supernumerary chromosomes in this species, besides addressing structural and morphological aspects, has also broadened the knowledge of aspects involved in the differentiation, evolution and relationships among species of the genus *Prochilodus*.

## Material and methods

In this study 29 individuals of the species *Prochilodus argenteus* (Table 1) resulting from crosses performed in the fish farm Projeto Peixe, Cravinhos – SP, Brazil, involving matrices derived from natural population of the São Francisco River, captured near Três Marias – MG, Brazil, were analyzed. The procedures for collection, maintenance and analysis of the fish were performed in accordance with the international protocols on animal experimentation followed by the Universidade Estadual Paulista. Morphometric and meristic data were taken following Castro and Vari (2004) and specimens were deposited at Laboratório de Biologia e Genética de Peixes fish collection, Universidade Estadual Paulista, Botucatu, SP. Vouchers of the used material are described in Table 1.

**Table 1.** B chromosome frequency and Mitotic Instability index (MI) of somatic cells in *Prochilodus argenteus*.

Specimen identification	Number of B per cell				MB	N	MI
	0B	1B	2B	3B			
4170	30	-	-	-	0B	30	0.000
4172	1	12	-	-	1B	13	0.157
4173	30	-	-	-	0B	30	0.000
4175	30	-	-	-	0B	30	0.000
4176	3	27	-	-	1B	30	0.200
4177	1	29	-	-	1B	30	0.034
4178	-	1	29	-	2B	30	0.036
4180	5	25	-	-	1B	30	0.337
4181	-	30	-	-	1B	30	0.000
4182	-	2	28	-	2B	30	0.065
4183	2	12	-	-	1B	14	0.294
4184	-	15	-	-	1B	15	0.000
4188	30	-	-	-	0B	30	0.000
4189	30	-	-	-	0B	30	0.000
4190	1	15	-	-	1B	16	0.133
4250	4	26	-	-	1B	30	0.274
4251	30	-	-	-	0B	30	0.000
4252	-	14	-	-	1B	14	0.000
4253	30	-	-	-	0B	30	0.000
4254	1	11	-	-	1B	12	0.173
4256	-	2	15	-	2B	17	0.111
4257	25	-	-	-	0B	25	0.000
4258	-	1	13	-	2B	14	0.072
4259	30	-	-	-	0B	30	0.000
4260	2	28	-	-	1B	30	0.136
4261	-	-	4	26	3B	30	0.082
4262	3	15	-	-	1B	18	0.337
4263	30	-	-	-	0B	30	0.000
4264	1	13	-	-	1B	14	0.152
						X_MI_	0,136

(MB) modal number of B chromosomes, (N) number of metaphases analyzed, (MI) Mitotic Instability index, (X_MI_) average MI among individuals with supernumerary chromosomes.

Chromosome preparations involved previous use of mitosis stimulation (Lozano et al. 1988, Oliveira et al. 1988) followed by cell suspension preparations using kidney tissue fragments of the individuals, according to the protocol proposed by Foresti et al. (1981). The karyotype organization was performed according to the method of Levan et al. (1964), using images processed by Adobe Photoshop CS5 program. To quantify the Mitotic Instability index (MI) the method of Pardo et al. (1995) was used. Active nucleolar regions (NOR) in metaphase chromosomes were identified by using silver nitrate staining (Howell and Black 1980) and the detection of constitutive heterochromatin (C-banding) was performed according to Sumner (1972).

Chromosomal mapping of ribosomal genes was performed with the technique of fluorescent *in situ* hybridization (FISH) according to Pinkel et al. (1986) using 5S and 18S rDNA probes obtained by PCR (Polymerase Chain Reaction) from genomic DNA of *Prochilodus argenteus*. Primers A (5_-TACGCCCGATCTCG TCCGATC-3_) and B (5_-CAGGCTGGTATGGCCGTAAGC-3_) (Pendás et al. 1994) were used to obtain the 5S probe, and NS1 (5_-GTAGTCATATGCTTGTCTC-3_) and NS8 (5_-TCCGCAGGTTCACCTACGGA-3_) according White et al. (1990), to obtain the 18S rDNA probe. The 5S probe was labeled with biotin-dUTP and the 18S probe was labeled with Digoxigenin-dUTP (Roche) by PCR, according to the manufacturer’s instructions. The preparations were stained with DAPI (4-6-diamidino-2-phenylindole) and examined in a fluorescence light microscope (BX 50, Olympus) equipped with an Olympus Q-color 5 digital camera. The photomicrographs were obtained using Q-Capture Pro 5.1.1.14 software.

## Results and discussion

**Figure 1.**
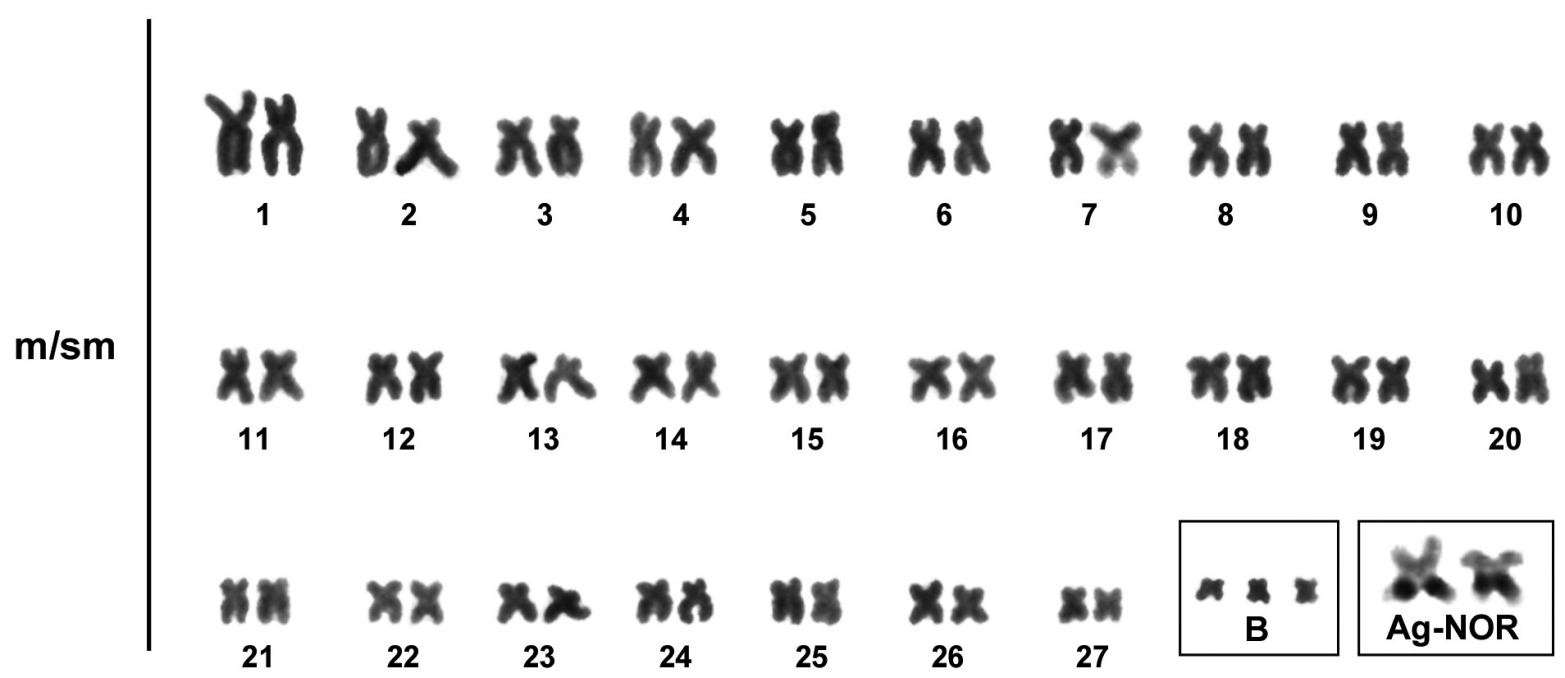
Karyotype of *Prochilodus argenteus* (2n=54 chromosomes). In the inset, three B chromosomes and the NOR bearing pair.

**Figure 2.**
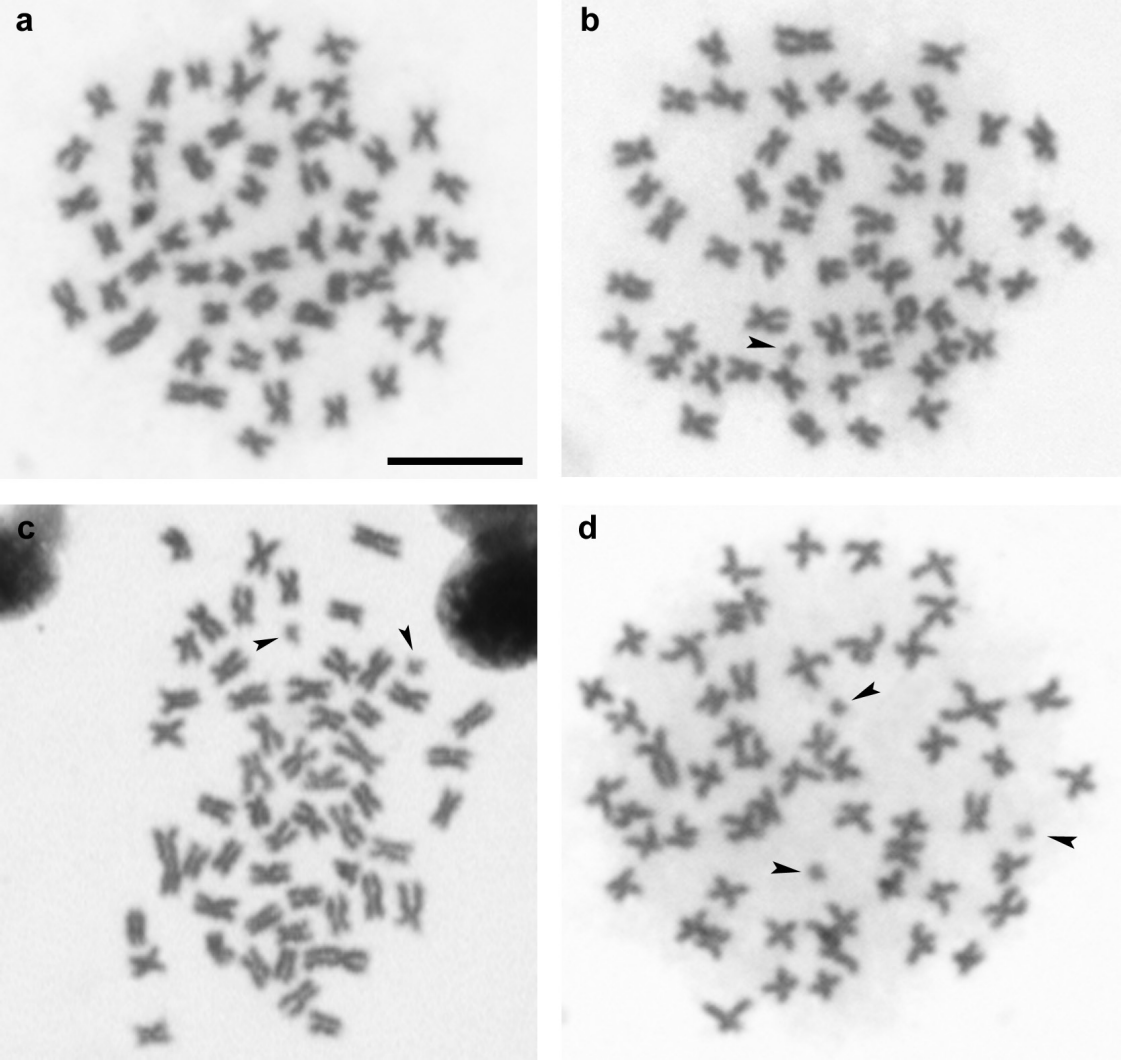
Metaphase plates of *Prochilodus argenteus* showing a cell of individual without B chromosomes (**a**); in individual presenting one B chromosome (**b**); cell presenting two B chromosomes (**c**); and cell presenting tree B chromosomes indicated by arrows (**d**). Bar = 10 µm.

Cytogenetic analysis performed on specimens of *Prochilodus argenteus* revealed the diploid number of 2n = 54 and fundamental number of 108 for this species, with a karyotype composed of meta and submetacentric chromosome types (Figure 1). No morphological differentiation between males and females was detected, confirming data published by Pauls and Bertollo (1983, 1990), Hatanaka and Galleti Jr. (2004) and, more recently by Voltolin et al. (2013). This karyotype identity, also present in other species already described is a conserved feature in the genus *Prochilodus*, and it may be identified also among the components of related groups (Galetti Jr. et al. 1994, Arai 2011). Among the 29 individuals analyzed the presence of up to three B chromosomes was observed, with a modal number in metaphases analyzed between zero and one B chromosome (Table 1, Figure 2), being that 14 individuals presented one B chromosome, four presented two B chromosomes and a single specimen carried three B chromosomes. Ten individuals were not carriers of supernumerary chromosomes in their cells in this sample.

One of the first descriptions of B chromosomes in Neotropical fish refers to additional chromosomes found in *Prochilodus lineatus* by Pauls and Bertollo (1983). Since then this species has been one of the most widely used in studies on the origin, inheritance and maintenance of these chromosomes in fish. Moreover, the occurrence of supernumerary chromosomes was also described in other species within the genus *Prochilodus* (Pauls and Bertollo 1990, Venere et al. 1999, Oliveira et al. 2003), adding to this list the species *Prochilodus argenteus* taken in this work. The number of studied species thus highlights this genus as one of the most representative and significant among Neotropical fishes concerning with the occurrence and study of these extra elements.

**Figure 3.**
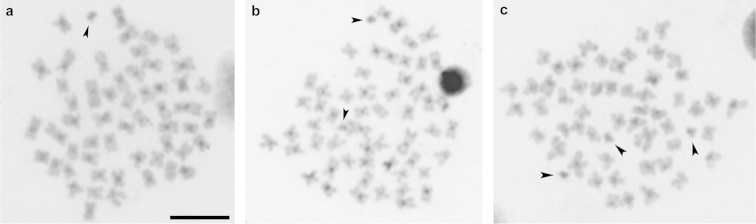
C-banding in metaphases plates of *Prochilodus argenteus* showing the distribution of heterochromatin in B chromosomes. Different cells showing one B chromosome (**a**); two B chromosomes (**b**); and tree B chromosomes indicated by arrows (**c**). Bar = 10 µm.

**Figure 4.**
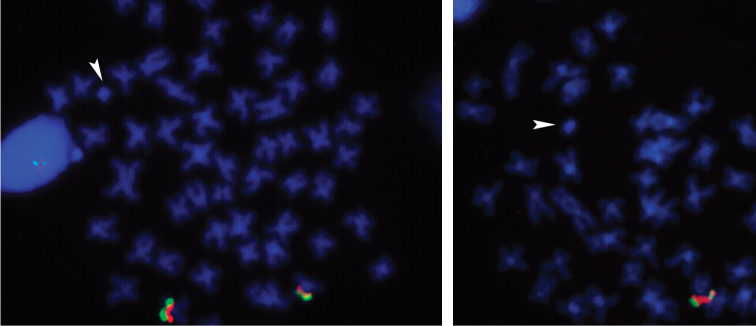
Fluorescent *in situ* hybridization using probes of 5S (green) and 18S (red) rDNA in metaphasic cells of *Prochilodus argenteus*, carrying one B chromosome (**a**) and two B chromosomes (**b**), indicated by arrows. FISH marked segments are visualized only on the NOR bearing chromosomes. Bar = 10 µm.

The supernumerary genomic elements found in this species can be easily identified in metaphasic cells due to their small size when compared with the chromosomes of the standard complement, usually heterochromatic, but presenting diverse and complex patterns of heterochromatin distribution (Figure 3). Such microchromosomes generally representing the metacentric type revealed no NOR mark after silver nitrate staining. Similarly, analysis of *in situ* hybridization using 5S and 18S rDNA probes did not reveal the presence of these genes in the supernumerary chromosomes (Figure 4). Visible signs of hybridization were observed in sinteny in one pair of chromosomes in the normal complement, as previously reported by Hatanaka and Galleti Jr. (2004) and Voltolin et al. (2013).

An interesting feature of B chromosomes present in the carrier species of this genus is the identification as completely or partially heterochromatic microchromosomes, which exhibit expressive inter and intra-individual number variation (Table 1). A higher frequency of variation is found in *Prochilodus lineatus*, which includes individuals without B chromosomes as well as others with up to nine supernumerary elements (Voltolin et al. 2011). In other carrier species the frequency of occurrence is less wide-ranging, with zero to three in *Prochilodus mariae* (Oliveira et al. 2003) and from zero to two in *Prochilodus brevis* and *Prochilodus nigricans* (Pauls and Bertollo 1990, Venere et al. 1999). It can be considered that the particularity of these genomic elements that do not follow the Mendelian segregation laws in meiosis could determine their independent nature, providing different ways of influence to their accumulation or disappearance during the evolutionary process.

The occurrence of homoplasy could be considered to explain the origin of morphologically similar genomic elements in species of the same biological group. Thus, the existence of specific chromosomes bearing structural elements capable of originating extra chromosomes in an ancestral form could act in an independent way and give rise to supernumerary chromosomes found today in some of these species. However, it can be also considered the idea of a common origin of these elements in all species of the genus from an ancestral carrier, followed by the loss in some species during the diversification process.

The rate of mitotic instability (MI) calculated from individuals with supernumerary revealed a mean value of 0.136 (Table 1). In studying the variation of MI in *Prochilodus lineatus*, Cavallaro et al. (2000) observed a decrease of 0.486 to 0.004 among individuals between the years 1979 to 1989, suggesting the occurrence of a stabilizing trend in the population studied. Comparison of the above results with the mean value of MI found in *Prochilodus argenteus* in the present study (MI = 0.136) permits the inference that the low values of instability in the sample analyzed in this work presents could be an indicative that the species is developing the stabilization of B chromosomes in somatic cells in the course of next generations.

The great similarity found in morphology, size, heterochromatic nature and frequency of B chromosomes of *Prochilodus argenteus* with those of other species in the genus *Prochilodus*, described as microchromosomes and, in general, totally heterochromatic (Pauls and Bertollo 1983, 1990, Venere et al. 1999, Oliveira et al. 2003, Voltolin et al. 2013) can identify patterns of origin and dispersion of these genomic elements in this group of organisms. The morphological and structural analyses of these extra elements in the genus *Prochilodus* could suggest a possible common mechanism of origin which would manifest independently in the species of this genus, and then would follow their own paths of differentiation and evolution.
